# Droplet and Particle Generation on Centrifugal Microfluidic Platforms: A Review

**DOI:** 10.3390/mi11060603

**Published:** 2020-06-22

**Authors:** Javid Azimi-Boulali, Masoud Madadelahi, Marc J. Madou, Sergio O. Martinez-Chapa

**Affiliations:** 1 School of Engineering and Sciences, Tecnológico de Monterrey, Ave. Eugenio Garza Sada 2501, Monterrey 64849, NL, Mexico; cavid.azimi@gmail.com; 2 Department of Mechanical and Aerospace Engineering, University of California Irvine, Irvine, CA 92697, USA; mmadou@uci.edu

**Keywords:** droplet generation, particle generation, bubble generation, multiphase flows, centrifugal microfluidics

## Abstract

The use of multiphase flows in microfluidics to carry dispersed phase material (droplets, particles, bubbles, or fibers) has many applications. In this review paper, we focus on such flows on centrifugal microfluidic platforms and present different methods of dispersed phase material generation. These methods are classified into three specific categories, i.e., step emulsification, crossflow, and dispenser nozzle. Previous works on these topics are discussed and related parameters and specifications, including the size, material, production rate, and rotational speed are explicitly mentioned. In addition, the associated theories and important dimensionless numbers are presented. Finally, we discuss the commercialization of these devices and show a comparison to unveil the pros and cons of the different methods so that researchers can select the centrifugal droplet/particle generation method which better suits their needs.

## 1. Introduction

Miniaturization and microelectromechanical systems (MEMS) gave birth to microfluidics in the 1990s [1]. The innovative paper by Manz et al. [2] established the field of miniaturized total chemical analysis systems for the first time [3]. Microfluidics is the science and technology dealing with integrated microscale channels (tens to hundreds of micrometers), through which small amounts of fluids (typically 10^−9^~10^−18^ L) can flow in specific fluidic structures in a controllable manner to systematically be manipulated and analyzed [4,5]. In microfluidics, since the geometrical dimensions are in microscale, fluid behavior is different from that of the macroscopic world as follows: the heat and mass transfer are more efficient, viscous forces are dominant over inertial forces, and surface effects are significant [6,7]. Moreover, the diverse coexistence of different fluid phases is easier in microfluidics due to the possibility of high integration [8]. Such characteristics are a driving force toward miniaturization and fluid control and manipulation in microscale. Therefore, microfluidics is a promising field of study for multidisciplinary research in biological [9,10], medical [11], engineering [12], chemical [13,14], and physical [15] fields. For instance, on-chip single-cell treatments [16]; optical fiber sensing [17]; fabrication of asymmetric lipid vesicles [18]; rapid chemical reaction monitoring [19]; direct loading of blood for plasma separation and diagnostic assays [20]; and fast and frugal diagnosis of malaria, sepsis, and HIV/AIDS [21] are some diverse recent advances in the field of microfluidics. Microfluidics encompasses the miniaturization, automation, and integration of laboratory processes ranging from basic operations to complex biochemical assays. It enables the development of low-cost and high-throughput bioanalytical devices for high-demand disease diagnosis and treatment. Microfluidic systems reduce experimental costs due to small sample volumes and required reagents and improve the accuracy of diagnosis by allowing multiple sample tests to be run in parallel. The compact size of microfluidic systems makes the point-of-care (POC) analysis possible, reducing the time required to obtain information about a sample.

Typically, lab-on-a-chip (LOC) devices consist of a network of microchannels, reservoirs, micropumps, and other components. Several kinds of actuation mechanisms are used for driving the fluid through the microfluidic circuit. In general, microfluidic pumps can be classified into the following two categories: displacement and dynamic micropumps [22]. Displacement micropumps apply pressure force on the working fluid through one or more moving boundaries, whereas dynamic micropumps continuously add energy to the working fluid by external body forces. Displacement micropumps can be subcategorized into reciprocating [23], rotary [24], and aperiodic pumps [25]. The majority of mechanical micropumps in the literature fall into the category of displacement micropumps because of less sensitivity to the working fluid. The membrane/diaphragm types operate when the oscillation of a thin membrane/diaphragm driven by actuators pushes the fluid forward. Although actuators such as piezoelectric mechanisms generate large actuation forces and have fast response times, they use a high electric voltage to operate which is sometimes not suitable for biological applications. Moreover, they have small strokes, the fabrication is complex due to the processing of piezoelectric material, the actuator has to run itself with chamber dead volume, they generate a pulsating flow rather than continuous flow due to their periodic nature, and the flow rectification, i.e., conversion to one-directional movement, necessitates using either microvalves (ball valves, check valves, etc.) or static structure valves such as nozzle/diffusers which adds to the complexity of the design and fabrication [26,27]. In general, mechanical moving parts such as microvalves and actuators in microfluidic systems are prone to wear and fatigue failure in the long run. In addition, the absence of moving components is desired specifically when the pumping fluid contains particles, such as cells, DNA samples, drug particles, etc. [27].

The use of external pumps such as syringe and peristaltic pumps are common in the field of microfluidics since they are easy to use. Generally, external pumps are expensive and each reagent in the assay requires a separate pump, which makes the experimental setup even more expensive [28]. These pumps are large and need a lot of external complex interconnects [28,29], which does not make them the best candidates for POC applications. In addition, since they are external pumps, they need long tubing systems for connecting to inlets and outlets, which requires high upstream pressures and wastes a lot of precious sample material as tubing dead volumes [30,31]. Additionally, the long narrow tubing systems hinder the processing of highly viscous fluids. Typically, syringe pumps offer more stable flows than other kinds such as peristaltic or recirculation pumps [32,33]. Although the mean flow rate can be very accurate when using syringe pumps, the transient flow is pulsatile and the pressure fluctuates overtime in the microfluidic chip [32]. At very slow flow rates, they show unsteady flow behavior in which the pressure can vary as much as 50% of the mean pressure value [34]. Additionally, syringe pumps have a long response time, which limits their use where dynamic flow profile studies are required in microfluidic systems [32]. Dynamic micropumps can be subcategorized mainly into centrifuge [35,36], electrohydrodynamic (EHD) [37,38], and magnetohydrodynamic (MHD) [39,40] pumps. Centrifugal pumping offers many advantages as compared with the EHD and MHD mechanisms which include the following: no need for conducting mediums [41]; ability to drive highly viscous fluids [42]; easy parallelization [28]; pulse-free [43] and bubble-free operation (which is common in EHD and MHD due to electrolysis [44,45]); and operation without parasitic heat induction which sometimes interfere with sensible pharmaceutical substances and biological samples [46].

In the present review paper, we focus on the generation of droplets, microparticles, bubbles, and fibers on centrifugal microfluidic platforms. We have presented all related applications, methods, and advantages of using centrifugal microfluidics, as well as relevant previous studies reported in the literature. We also compare different methods to highlight the pros and cons of each technique and present a short discussion on the recent commercialization status.

## 2. Benefits of Using Droplets, Particles, Bubbles, and Fibers

According to what is needed on a microfluidic platform, it could include fluids carrying an immiscible material (which can be solid-like particles and fibers, fluid-like droplets, or gas-like bubbles). The benefits of using droplets, particles, bubbles, or fibers that can help scientists on a microfluidic device are:

1. Droplet microfluidics involves the production and manipulation of discrete droplets in the scale of nanometer to micrometer at rates of up to about four thousand droplets per second [47]. Since the surface area to volume ratio is high for microscale droplets, diffusion distances are small. Hence, the time needed for heat and mass transfer is short, and chemical reactions are quick [48].
2. Additionally, sample, cell, or reagent encapsulation in droplets ensures no direct contact between samples and microfluidic surfaces, securing high purity of reactions [49].
3. Moreover, droplet-based microfluidics offers independent control of each droplet, which can be individually produced, transported, and analyzed, offering precise control of the solutions [48].
4. It is proven that secondary flows in small scale size can help to mix the ingredients within the droplets significantly [50,51].

## 3. Applications of Droplets, Particles, Bubbles, and Fibers

According to the benefits mentioned above, scientists can use droplet, particle, bubble, or fiber generation units in their microfluidic devices for a specific application. These benefits exist for any microfluidic device including centrifugal or non-centrifugal. As a case in point, if a scientist aims to carry a small volume of sample without any probable contamination while touching the walls of the microfluidic device, they can discretize it into small sample droplets in a carrying fluid flow. Madadelahi et al. used a new fabrication technique to fabricate high-pressure microfluidic devices. They used chloroform and paraffin to attach different layers of PMMA sheets. However, since the surfaces of microchannels were not completely smooth and clean, they put the genes into discretized droplets to avoid them touching the microchannels surfaces. Using this technique of droplet-based fluid flows, they were able to study the effect of nanodiamond particles on gene amplification [51]. Many such applications for droplets, particles, bubbles, and fibers are as follows:

Droplet: Droplet generation is one of the basic functionalities of microfluidic devices. Droplets are useful in absolute quantification of L. monocytogenes in food production [31], digital counting of colony-forming units (CFUs) [52], monodisperse particle production for controlled release of medical solutions [53,54], development of droplet-based POC diagnosis [55], culture assays, cell encapsulation, single-cell analysis at ultrahigh-throughput [56,57,58], and enzyme evolving with enhanced function [59,60]. Since droplets can isolate samples in numerous partitions, they are beneficial in digital assays such as culture-based screening, digital enzyme-linked immunosorbent assay (ELISA), loop-mediated isothermal amplification (LAMP), multiple displacement amplification (MDA), strand-displacement amplification (SDA), rolling circle amplification (RCA), recombinase polymerase amplification (RPA), and polymerase chain reaction (PCR) [61,62]. Digital PCR (dPCR) is a specific PCR in which DNAs are distributed into a large number of isolated water-in-oil (W/O) emulsions. This is a promising method for precise measurement of nucleic acids offering some advantages over bulk reaction, including absolute quantification without the need for standard curves, as well as improved sensitivity and accuracy, which enables the analysis of isolated molecules and cells [63]. dPCR has many medical applications such as detection of pathogens, highly accurate quantification of gene expressions, and minority alleles or copy number variations [64,65]. Moreover, W/O emulsions are widely used for other assays such as isothermal amplification methods [66], and cell-based assays [47,67,68].

Particles: Polymeric particles which are made of natural polymers are of significant interest in biological and paramedical studies because of their biocompatibility, abundance, and low cost of natural polymers. They are widely used in the encapsulation of biomolecules, scaffolding of cell cultures, and controlled releases of biomaterials [69]. As a protection layer, coating of a solid or liquid phase with a layer is the primary goal of encapsulation. Some applications of microparticles include encapsulation of vitamins, minerals, or even probiotic bacteria in the food industry [70]; colors and flavors in the cosmetic industry; encapsulation vehicles for pharmaceuticals [71], cells [42], enzymes, and proteins in drug delivery [72]; and compartments for cell-sized functional microbeads [73]. For diffusively mass transport, in these applications, monodispersity and being small in size are critical factors [42].

In addition to the applications of ordinary particles, Janus particles made of at least two portions (with different chemistry or polarity) have many applications which include the following: fabrication of bi-colored beads for display technologies and switchable display panels [74], optical probes for rheological measurements or biological interactions in confined space [75], increased diffusion coefficient as compared with standard particles in nanomedicine owing to self-propulsion capability by catalytically active Janus particles [76,77], and efficient dispersion of oil contaminants in seawater [78] (combined capabilities of particulate emulsifier and chemical surfactants with amphiphilic Janus particles possessing both hydrophobic and hydrophilic sides). As another application, magnetic Janus particles are an attractive vehicle for controlled drug delivery purposes. Magnetic materials and biological species can be encapsulated in each hemisphere to avoid interaction between them. Magnetic hemisphere makes the target drug delivery based on the magnetic control possible, while the other hemisphere can be used as a carrier for biological species and pharmaceuticals [79].

Bubble: Bubbles have a minor difference as compared with droplets; they are hollow structures made of a thin layer of liquid. Microbubbles have a broad scientific and technological importance mainly in applications related to the targeted drug delivery [80,81], food processing [82], heavy metal separation in mineral processing [83], ultrasonic imaging [84], controlled release of chemicals [85], and development of bubble base logic circuits [86].

Microfibers: Together with the abovementioned structures, microfibers can be generated in many droplet/particle generation devices. The change of droplet to fiber production occurs by changing the flow regime in a droplet/particle generation device (different regimes are explained in the next sections). Micro threads or fibers have found emerging importance in applications such as optical sensors and producing hydrogel microfibers for cell scaffolding and precise control of cell orientation, which is essential for specific cells and appropriate functionalities of organs and tissues [68,87].

Among the different mentioned applications in the above sections, POC is one of the most important applications. In the next section, the advantages of centrifugal microfluidics are mentioned. This reveals that centrifugal microfluidics can be considered to be one of the most suitable platforms for POC applications in resource-limited areas in which primary care is insufficient for living [88].

## 4. Active Droplet Generation Methods

Although the majority of previous works have used syringe pumps for passive droplet/particle generation, they can also be actively produced and manipulated by forces such as electrical, magnetic, and centrifugal forces. In comparison with passive methods, these forces offer easy handling and higher flexibility in controlling the droplet size and production rate because of short response time, and easy adjustability [89].

In order to produce droplets with electric fields, electrodes have to be embedded within microfluidic devices, which are connected to high electric voltages. According to the current type, the electric field can be applied in two ways, i.e., direct current (constant or pulsatile DC voltage) [90] or alternating current (low-frequency and high-frequency AC voltage) [91]. Numerical simulation results based on the DC control, show three distinct stages of droplet size variations. By increasing the electric voltage, the droplet size reduces at first, then grows, and again decreases, which is explained by counterbalance between the components of electrical forces [92]. However, when a pulsatile DC is applied, the phase interface moves into a main narrow channel by electrical forces. Nevertheless, the secondary droplets form because of Rayleigh’s instability [93,94]. In low-frequency AC control, the droplet size is affected by the measure of the electric field, but it is independent of the polarity. Additionally, droplet production is asynchronous with the applied electric field and shows hysteresis [95]. Tan et al. identified three regimes, i.e., dripping, unstable droplet production, and jetting by systematically changing the voltage frequency and electrical conductivity of the dispersed phase [89,91].

A magnetic field as an external force can be used to generate droplets where a ferrofluid is used as the dispersed phase. Ferrofluids contain magnetic nanoparticles, which are magnetized and demagnetized by applying and withdrawing external magnetic fields [89]. The magnetic field can be created by permanent magnets [96] or electromagnets [97] covering fully or partially the microfluidic device and applied uniformly [98] or nonuniformly [96]. Proper implementation of the magnetic field is necessary for droplet generation. First, the orientation of the magnetic field is important. In addition, changing the position of permanent magnets varies the orientation and uniformity of the magnetic field strength. In the T-junction configuration, for example, the droplet size decreases when the magnet is placed downstream, whereas the size increases when the magnet is positioned upstream [99]. Additionally, in the flow-focusing configuration, magnetic fields, perpendicular and parallel to the fluid flow, influence the expanding and breakup process of the dispersed phase, respectively [98]. Moreover, the device geometry is an effective parameter in modulating the droplet size. In the case of the uniform magnetic field parallel to the main flow, as magnetic flux density grows, the droplet size in flow focusing increases; in contrast, it decreases in T-junction geometries [89,100].

Recently, the artificial gravitational force in centrifugal microfluidics has been widely used because of several advantages over two other mentioned methods. The reasons for this case are the following:

- First, unlike traditional microfluidics that need complex interconnections for several external pumps, centrifugal force exists in every part of the rotating assay, and then sample propulsion capability is available. It provides actuation for all microfluidic structures simultaneously. In addition, the scalability of the centrifugal systems is very high since there is no need for other external pumps for multiple parallel operations [28].
- Second, centrifugal systems are capable of processing highly viscous fluids. Since the diameter of the nozzle is an essential parameter in the generated droplet size, producing small droplets with conventional methods puts severe challenges on the fabrication of small nozzles. Although using pressure pumps increases throughput to some extent, it changes the dynamics of dripping flow to undesired flow regime. However, applying high centrifugal force can provide a strong force to induce highly viscous multiphase flows. Moreover, since encapsulating biological samples and cells into small droplets requires bigger nozzles, the centrifugal systems provide additional forces to pinch-off the small size droplets [42].
- Third, centrifugal body force linearly increases with the radial distance from the center, providing pressure distribution inside the channel below the (atmospheric) pressure in the inlet and outlet of the channel. This unique pressure distribution offers high flow rates and throughput without complicated requirements for channel sealing [101,102].
- Fourth, unlike magnetic and electric propulsion systems, which are only applicable to Ferro and conductive fluids, centrifugal pumping is independent of the working fluid’s properties such as electrical, magnetic, and chemical properties. In addition, applying electric fields sometimes causes electrolysis and forms bubbles, which is detrimental for microfluidic systems [29].
- Fifth, centrifugal systems tend to dampen high-frequency pressure fluctuations by their angular momentum, resulting in a pulse-free flow and enhanced reproducibility of the pinch-off process [46].
- Sixth, this approach is simple and robust in the hands of non-experts. A simple rotating motor with a control and a microfluidic device is enough to produce droplets/beads. There is no need for several expensive syringe pumps and high voltage power supplies [29].

## 5. Theory

Identifying the most important parameters affecting the flow is the key to better control and manipulate microfluidic devices. Dimensionless numbers are beneficial tools for characterizing relative predominance of different effects in the fluid flow.

In centrifugal systems, there are pseudo forces comprising the centrifugal force (**F**_c_) (Equation (1)), Coriolis force (**F**_cor_) (Equation (2)), and Euler force (**F**_E_) (Equation (3)). These are body forces exerted on the mass (*m*) at every specific position on the centrifugal platform [103].

(1) $$F_{c}=-m\omega \times \left(\omega \times r\right)$$

(2) $$F_{\mathrm{Cor}}=-2m\omega \times \frac{dr}{dt}$$

(3) $$F_{E}=-m\frac{d\omega }{dt}\times r$$

where **ω** is the rotating velocity, *t* is the time, and **r** is the distance from the rotating center. The droplet formation process can be categorized into the dripping regime and the jetting regime. The transition between dripping and jetting regimes can be estimated by the dimensionless capillary number (Ca) of the continuous phase and the Weber number (We) of the dispersed phase. The Ca number is defined as the ratio of viscous to surface tension forces and the We number is defined as the ratio of inertial to surface tension forces [89,104]. Ca and We numbers can be expressed as follows:

(4) $${\mathrm{Ca}}_{\mathrm{CP}}=\frac{\eta_{\mathrm{CP}}u_{\mathrm{CP}}}{\gamma }$$

(5) $${\mathrm{We}}_{\mathrm{DP}}=\frac{\rho_{\mathrm{DP}}u_{\mathrm{DP}}^{2}L}{\gamma }$$

where u_CP_ and u_DP_ are the velocities of the continuous and dispersed phases, respectively; *γ* is the interfacial tension between two phases; *η* is the viscosity; *ρ* is the density; and *L* is the characteristic length scale of the microfluidic channel.

Droplets are generated at a dripping regime where surface tension forces dominate, and therefore Ca and We are small. However, large values of Ca and We numbers indicate a jetting flow regime and the dominance of viscous and inertial forces, respectively. The transition from the dripping regime to the jetting regime occurs at a critical capillary number Ca_Cri_, which is experimentally discovered to be between 0.023 and 0.050 [47,105].

Centrifugal microfluidic platforms can produce droplets by intrinsic artificial gravitational forces, including centrifugal, Euler, and Coriolis. The dimensionless Bond number (Bo) (Equation (6)) quantifies the impact of centrifugal acceleration, representing the ratio of centrifugal force over surface tension force.

(6) $$\mathrm{Bo}=\frac{\Delta \rho gL^{2}}{\gamma }$$

where Δ*ρ* is the density difference between dispersed and continuous phases, and *g* denotes the artificial gravitational force. Droplet size and production rate can be adjusted by controlling the Bo number, which can be achieved by changing the rotational speed and channel geometry [104].

## 6. Different Methods of Droplet/Particle Generation on Centrifugal Platforms

**Figure 1:**
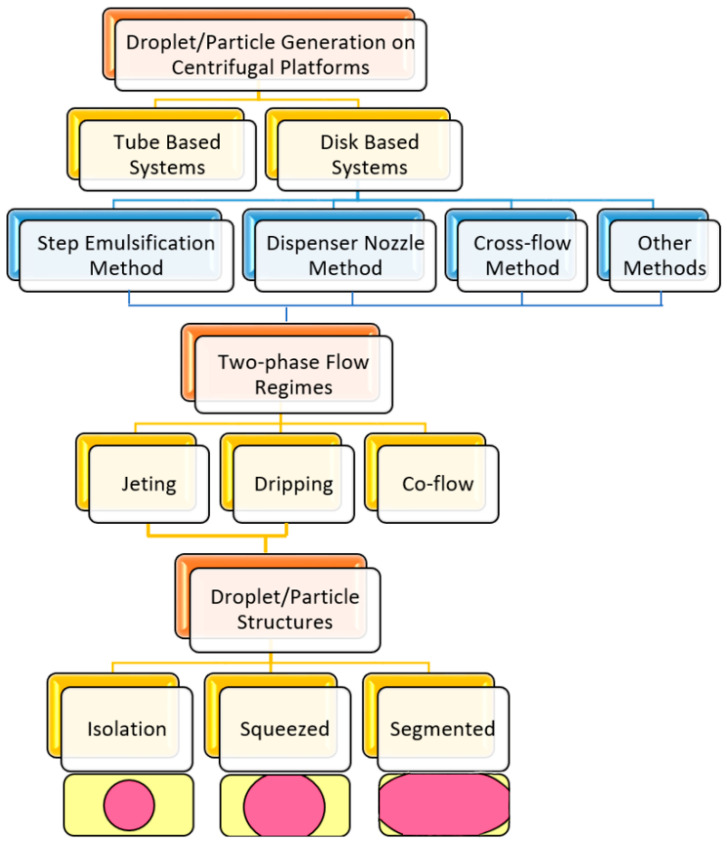
Different methods of droplet/particle generation on centrifugal microfluidic platforms.

Generally, there are two types of centrifugal microfluidics, i.e., tube-based devices and disc-based microfluidics. On the one hand, tube-based devices are easy to use and compatible with biomedical protocols because commercial centrifugal and standard microtubes are adapted to this method. On the other hand, disc-based platforms are flexible and affordable for innovative configurations. For both of these two platforms, different methods for droplet/particle generation can be categorized into four groups. As depicted in Figure 1, step emulsification, dispenser nozzle, and crossflow are the main three categories. In addition, a few minor novel methods exist in the literature, which are considered as “other methods”. In the following sections of this review paper, we introduce each method in detail, then, together with the previous studies, we characterize and finally compare them. From the fluidic point of view, three main two-phase flow regimes are possible, including jetting, dripping, and co-flow [106]. Among two possible methods (jetting and dripping) for making discretized droplets, three situations are possible for the structures. As shown in Figure 1, when the size of the spherical droplets is smaller than the channel height and width, the structure is called isolated. When the relaxed diameter size is smaller than the channel width but larger than the channel height, the structure is called squeezed (useful for the production of non-spherical microparticles [107]). When the relaxed diameter size is bigger than both channel height and width, the structure is called segmented flow (they are interesting for analytical applications [108]).

### 6.1. Step Emulsification Method

**Figure 2:**
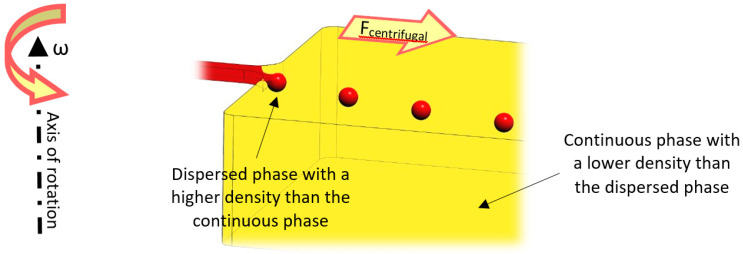
A schematic view of the step emulsification method.

Step emulsification is one of the common methods in droplet and particle generation in lab-on-a-disk (LOD) devices. In this method, only one channel is required for the droplet formation. As shown in Figure 2, the droplets are formed due to the abrupt change in capillary pressure when the flow passes through a channel with a backward-facing step. Design, fabrication, and control of fluid flow for only one microchannel, make this method very simple and robust as compared with the T-junction and crossflow systems (to be described afterward). Additionally, this method is capable of generating high-volume fraction (the ratio volume of dispersed phase to total volume of the emulsion) emulsions, while fluctuations in both flow rate and pressure do not have a significant effect on the monodispersity [109]. Moreover, since this method produces droplets without exerting high shearing forces by the continuous phase, this method is a good option for some applications where biological samples and cells are encapsulated in droplets [46,110]. Parallelization is quite easy in this method because the fluid flow in parallel nozzles can be controlled only by adjusting rotational speed. However, the accumulation of droplets in the nozzle tip is a disadvantage which can limit the high-throughput capability of these systems [31].

**Figure 3:**
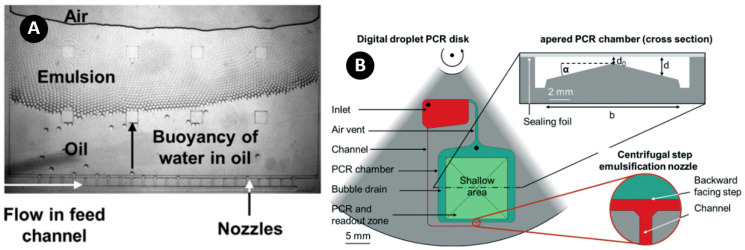
(**A**) A microscope image of multiple nozzles producing homogeneous droplets. Reprinted with permission from [31]; (**B**) Step emulsification and bubble removal design in PCR chamber. Reprinted with permission from [111].

As shown in Figure 3A, Schuler et al. found the step emulsification method to be an easy and fast way to produce monodisperse droplets [31]. They proved the robustness of the centrifugal approach by demonstrating that the droplet size was independent of the dispersed phase flow rates between 0.01 and 1 µL/s. This method was used for digital droplet recombinase polymerase amplification (ddRPA) and it was shown that the overall time for the process could be reduced by more than a factor of four (from 2 h to 30 min) as compared with the digital droplet polymerase chain reaction (ddPCR). The same team, in another work, presented an integrated system combining emulsification, PCR, and fluorescence readout in a single chamber [111]. The specific chamber design (Figure 3B) allowed the removal of gas bubbles generated during the PCR thermocycling through tapered regions in the shallow chamber. They reported that this method was effective and efficient based on the fact that 100% of the bubbles were removed by capillary force, and no merging of droplets due to shear forces was observed.

**Figure 4:**
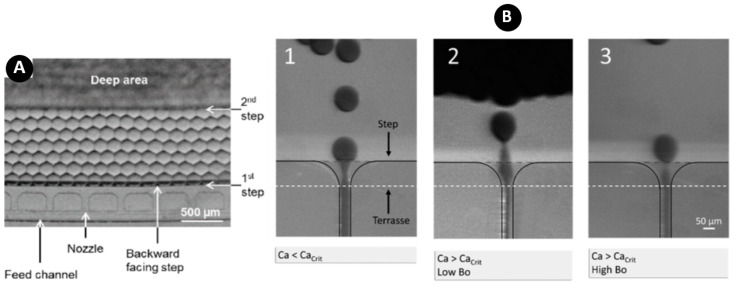
(**A**) Centrifugal force packs droplets together to form a hexagonal pattern by removing excess oil, and consequently, achieving high-volume fractions [109]; (**B**) Stroboscopic images of the detachment process at the nozzle for the dripping and jetting regimes in centrifugal step emulsification in which high buoyancy at high Bo numbers support rapid breakup process and monodisperse droplets. Reprinted with permission from [47].

Schuler et al. showed the capability of generating high-volume fractions above 95% with centrifugal step emulsification [109]. In contrast to their previous work, they arranged the nozzles in eight rows of nine nozzles for high-throughput demands, producing 3700 droplets per second. As shown in Figure 4A, centrifugal force helped the droplets become packed together and removed the excess oil resulting in high-volume fraction. However, reaching high-volume fractions was difficult, for example, when they attempted to reach 99% of volume fraction at 20 Hz, the droplets close to the center of the disc merged and formed a water layer. In addition, by reducing the rotating speed by half (10 Hz) to avoid merging, the buoyancy of the droplets reduced, and they did not pack densely. Hence, more oil was trapped between the droplets, which resulted in a low-volume fraction. Moreover, droplet accumulation near the nozzle tip at low rotating speeds prevented the production of new droplets and resulted in inhomogeneous droplet sizes. In a similar work, Schulz et al. studied the effect of buoyancy on the droplet formation rate with a step geometry [47]. They found that buoyancy could be utilized in producing droplets above the critical capillary number. In other words, because buoyancy facilitated the droplet formation, it was possible to produce monodisperse droplets in the dripping regime without compromising the diameter and CV (coefficient of variables, that is, the ratio of the standard deviation of the diameter to the average diameter of the droplets/particles). They achieved a rate of 2800 droplets per second per nozzle for producing droplets with a diameter of 100 µm. This enabled the emulsification generation without the need for parallelization in high-throughput applications where only one single nozzle was available. In cases where parallelization was needed, the required number of nozzles would be much less, for example, using only 36 units generated above 100 K droplets per second. Figure 4B depicts the process of droplet formation in different Bond numbers in which high buoyancy helped the successful breakup process.

**Figure 5:**
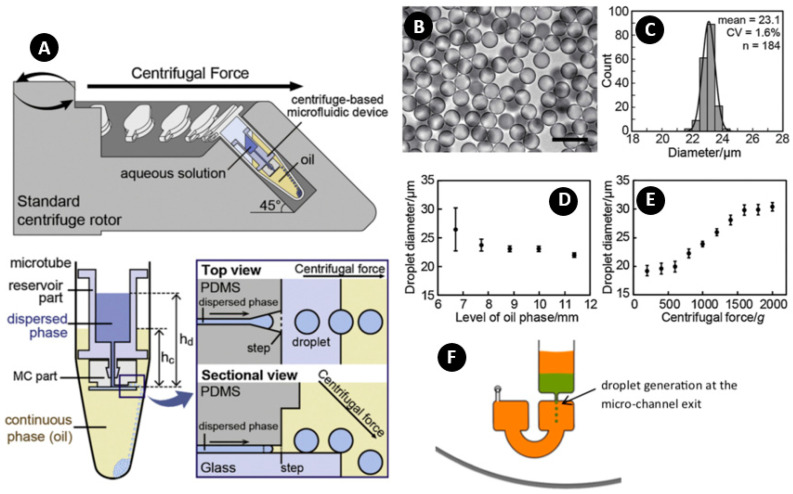
(**A**) Principal of step emulsification in a centrifugal system. (**B**) Microscope image of produced droplets at 1000 g (scale bar 50 µm); (**C**) Diameter distribution of the produced droplets; (**D**) Effect of the level of oil phase to droplet diameter; (**E**) Effect of centrifugal force to droplet diameter. Reprinted with permission from [73]; (**F**) Droplet generation in stationary carrier phase. Reprinted with permission from [43].

Shin et al. used a triangular shaped gradually expanding channel and a step at the end (Figure 5A) for inducing step emulsification [73]. The setup was run with a commercial centrifuge system with a fixed angle of 45° for the simple and fast generation of monodisperse picolitre droplets. Since the density of the continuous oil was less than the water, the droplets sank to the bottom of the tube after formation. Microscope image (Figure 5B) and diameter distribution (Figure 5C) of the produced droplets indicated a good monodispersity (CV = 1.7) and picolitre size (diameter = 23 µm). In this system, the monodispersity of the droplets depended on the level of the dispersed phase and, at low levels of the oil phase, the droplets became polydispersed (Figure 5D). In addition, if the centrifugal force was not high enough as compared with the droplet generation, the droplets accumulated and merged at the exit of the channel resulting in polydispersity. As shown in Figure 5E, the droplets’ diameter was independent of the centrifugal force in low rotational speeds [112]. However, with an increase of rotational speed, the droplets’ diameter linearly increased, and then saturated to a limit maintaining its monodispersity. This robust monodisperse droplet generation at high centrifugal forces has not been observed in conventional step emulsifications [31]. Moreover, in a constant aspect ratio of the channel, the bigger the channel height, the bigger the droplets. Soroori et al. were the only group that added a supportive fluid behind the dispersed phase liquid [43]. As shown in Figure 5F, the sample liquid (green liquid) was sealed from the top and bottom by polybutene layers (orange liquid). This method had some advantages over the moving carrier phase such as smaller shear force (high shear forces can be detrimental for biological samples and cells), independency of shearing forces on the remaining liquid, ability to produce continuous droplets for an extended time, and preventing the evaporation of the sample liquid by sealing it from the top and bottom during the thermal cycling.

We have summarized all the characterizations of step emulsification studies in Table 1. These characteristics include diameter range, CV, volume fraction, production rate, and rotational speed/acceleration.

### 6.2. Dispenser Nozzle Method

**Figure 6:**
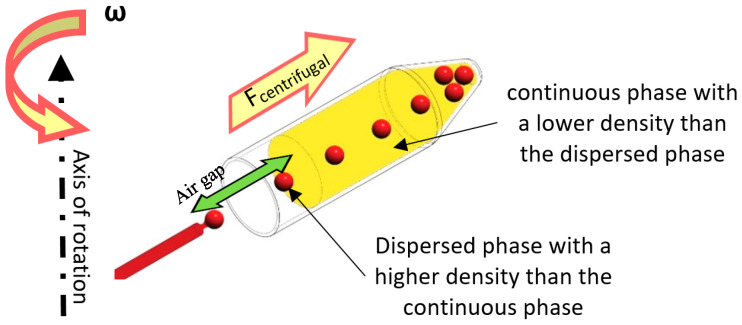
Schematic view of the dispenser nozzle method for droplet and particle generation. Droplets are produced at the tip of the nozzle and enter a tube containing a continuous phase.

Use of natural polymer particles is becoming more and more popular because they are abundant, low cost, and biocompatible. Hydrogels and microbeads have numerous applications in pharmaceutical engineering and biomedical science such as encapsulation of biomolecules, controlled release of drugs, and scaffolding. Figure 6 illustrates the dispenser nozzle method where droplets are generated at the nozzle tip. Then, they travel through an air gap and enter a continuous phase to be crosslinked and collected.

Equation (7) estimates the droplet diameter (*d_drop_*) with the nozzle diameter (*d_n_*), artificial gravitational force (*g*), density (*ρ_drop_*), and surface tension (*σ_drop_*) of the liquid. For producing small droplets, according to Equation (7), scientists need to overcome the manufacturing challenges to fabricate small nozzles [42]. Using small nozzles decreases the production rate. To overcome this issue, we can increase the pumping pressure to some extent; however, it changes the flow dynamics from dripping to the jetting regime, which is undesirable. More seriously, in some applications where encapsulation is needed, cells and big particles do not fit easily through very tiny nozzles. Centrifugal force (higher g values) enables the use of bigger nozzles for the production of microdroplets for cell encapsulation purposes. Moreover, centrifugal force enables the processing of highly viscous fluids (higher *σ*) to produce small particles [42].

(7) $$d_{drop}=\sqrt[{3}]{\frac{6d_{n}\sigma_{drop}}{\rho_{drop}g}}$$

Nozzle clogging is a common issue when the nozzle comes into direct contact with the crosslinking solution. To overcome this issue, one method is to disperse the continuous phase into an immiscible oil phase [114]; however, this method is slower because it needs additional washing steps of the beads. A more straightforward technique is to use an air gap between the nozzle tip and the continuous phase, which prevents direct contact with the crosslinker solution and is faster and without cross-contamination [42,115]. However, there is a risk of droplet bursting when flying droplets touch the viscous liquid surface after traveling the air gap at high rotational speeds. Therefore, the low viscosity of the continuous phase is preferred in this method to avoid droplets being smashed when passing through the interface. Another limitation is that the density of the continuous phase has to be lower than the dispersed phase to let the formed droplets sink to the bottom of the tube. However, the density difference between the dispersed phase and continuous phase needs to be small, providing enough buoyancy to ensure droplets do not merge together.

**Figure 7:**
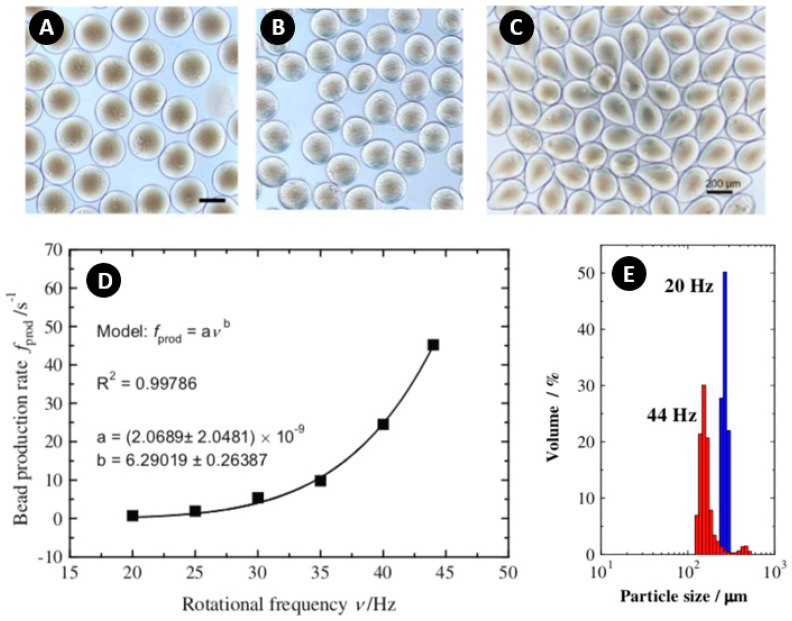
(**A**–**C**) Microscope images of beads. (A) 2% uniform particles of chitosan at 20 Hz; (B) 2% particles of chitosan at 25 Hz, smaller droplets retaining spherical shape; (C) 3% tear-like shape chitosan at 30 Hz; (**D**) Bead production rate at different rotational frequencies; (**E**) Size distribution histogram at 20 and 44 Hz with 2% chitosan. Reprinted with permission from [115].

For the first time, Mark et al. produced chitosan beads using dispenser nozzles on the centrifugal platform [115]. In their configuration, the beads’ diameter changed between 148 and 257 µm by controlling the rotational frequency. An increase in the viscosity of the chitosan solution and rotational speed resulted in a tear-like shape of the beads, which was attributed to the low tendency of the drops to regain their spherical shape because of the high viscosity and fast gelation at high rotating speeds (Figure 7A–C). Because of the non-Newtonian, shear-thinning behavior of the chitosan [116], the bead production rate approximated to the sixth power of rotational frequency according to the experimental results (Figure 7D). Additionally, during the gelation process, droplets shrank in diameter with a ratio of 0.69. Moreover, Figure 7E shows that the produced beads at low frequencies were more monodisperse and bigger as compared with the beads produced at high frequencies.

**Figure 8:**
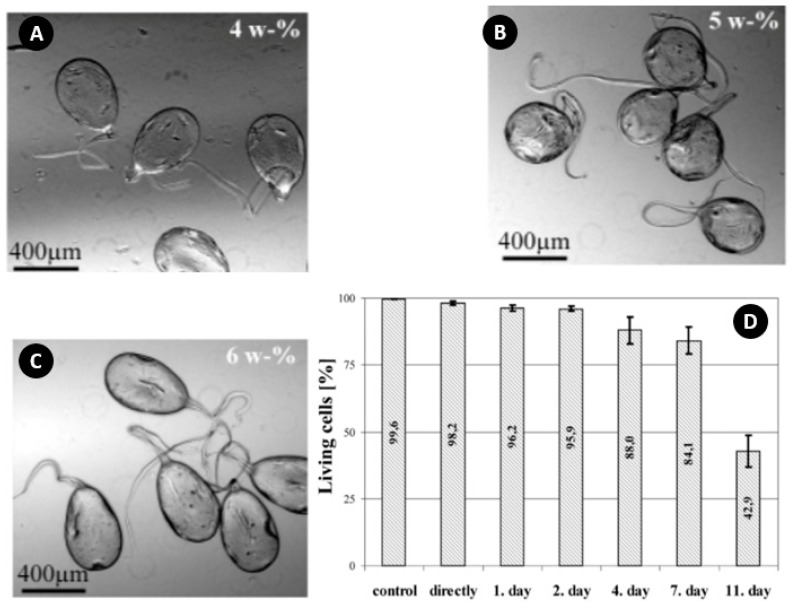
(**A**–**C**) Microbeads produced by different concentrations (4, 5, and 6 wt%) of Na-alginate solution; (**D**) Cell vitality before and after encapsulation (PC12). Reprinted with permission from [42].

Haeberle et al. used highly concentrated Na-alginate solutions (with a viscosity of 50,000 times more than water) to create microbeads and encapsulate two different cell types of PC12 and HN25, into single Ca-alginate beads [42]. In their study, experimental results with different concentrations of Na-alginate (4, 5, and 6 wt%) and nozzle sizes of 511 µm and 562 µm, at a frequency of 40 Hz showed a growing tail and nonspherical shape of droplets (Figure 8A–C). The formation of these tails was attributed to the hardening of the bridge during the droplet formation. According to Figure 8D, a cell vitality test for PC12 cells showed that the majority of the cells were still alive after two days, and a significant decrease of vitality was not observed until the fourth day (88% down to 42.9% on Day 11).

**Figure 9:**
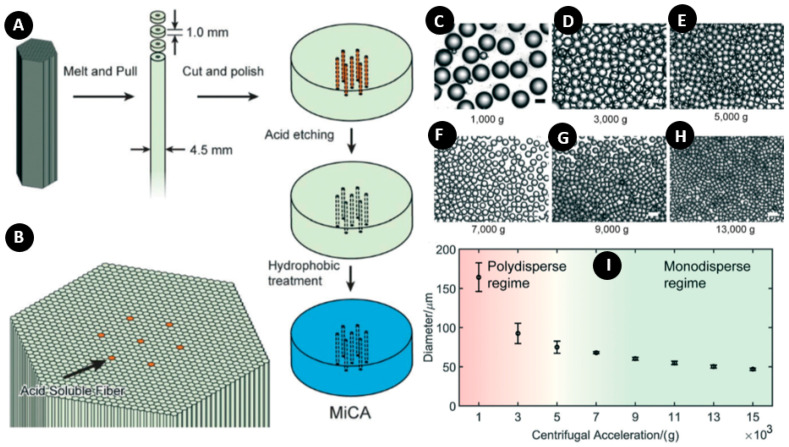
(**A**) Microchannel array (MiCA) fabrication and characteristics; (**B**) Acid-soluble fibers within the bundles; (**C**–**H**) Microscope images of the produced droplets in various centrifugal accelerations; (**I**) Morphology and size of droplets generated at various centrifugal accelerations. Reprinted with permission from [64].

Chen et al. used a microchannel array (MiCA) for the production of water-in-oil emulsions [64]. In comparison to PDMS and glass-based microfluidic platforms, the fabrication of MiCA plates can be cost-effective due to the massive production capacity of the microchannel plate technique. In this method, the dispersed phase passed through several microchannels equidistantly spaced. This parallelization promoted the production rate and decreased the probability of total clogging. Figure 9A shows the fabrication and characterization of MiCA in which two types of glass fibers are used to form a hexagonal lattice symmetry (Figure 9B). Generally, smaller holes with higher centrifugal forces favor the production of high-quality small W/O emulsions. The researchers in this study found that the optimal hole size was 6 µm. An air gap between the MiCA and the continuous phase prevented loss of material, cross-contamination, and clogging of the microchannels. To avoid droplet smash, a mixture oil with a density of 0.85 g cm^−3^ and a viscosity of 12 cSt was used. It was proven that this oil was fully compatible with the PCR buffer and helped the stabilization, compartmentalization, and heat transfer for ddPCR. The results indicated that droplets became smaller and more monodispersed by increasing the rotational speed (Figure 9C–H), and the transition between polydispersity and monodispersity fell around 5000 g (Figure 9I).

**Figure 10:**
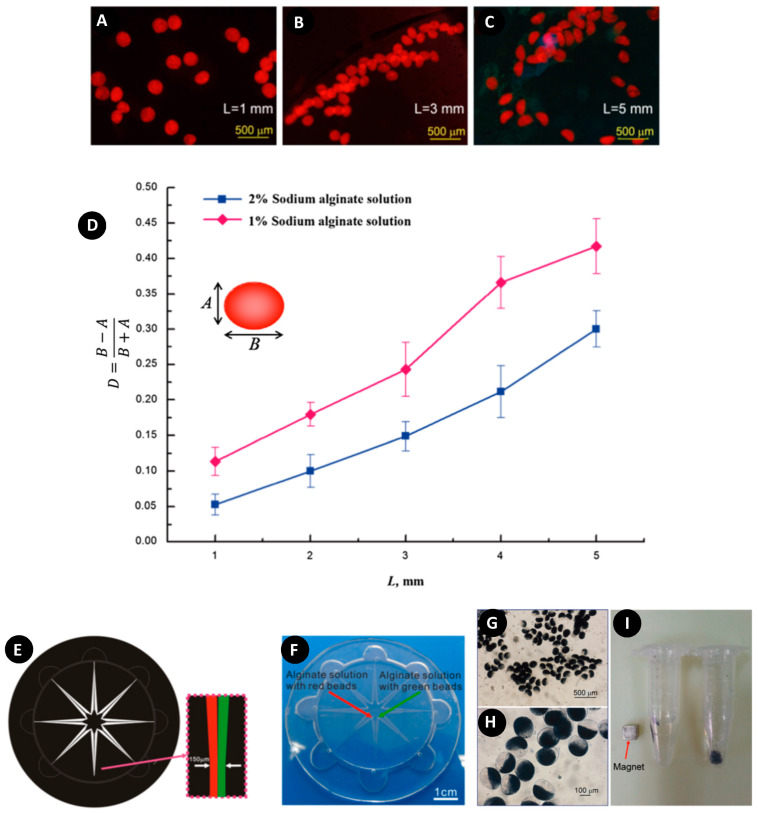
(**A**–**C**) Fluorescence images of produced polymeric particles by adjusting the airgap (L); (**D**) Effect of the airgap distance (L) on the deformation of the particles (D) in different Na-alginate concentrations; (**E**,**F**) Production of Janus particles on centrifugal microfluidics; (**G**,**H**) Production of magnetic Janus particles; (**I**) Manipulation of magnetic Janus particles with a magnet. Reprinted with permission from [79].

Liu et al. used a novel approach to produce oblate spheroidal Ca-alginate particles using centrifugal force [79]. In this method, the dispersed phase pinched off from the channel instead of a nozzle to enter the air gap. As depicted in Figure 10A–C, the air gap size had an effect on the shape of the produced particles; the longer distance droplets fly, the larger the deformation they undergo. This was attributed to the influence of air stress during the travel period. In addition, Equation (8) was used to quantify the deformation amount from a spherical shape to study the effect of Na-alginate concentration on the generated beads [117].

(8) $$D=\frac{B-A}{B+A}$$

where *A* and *B* are the minor and major axis of the deformed beads, respectively.

As illustrated in Figure 10D, droplets of 1% Na-alginate solution experience larger deformation as compared with those of 2% Na-alginate solution, which was because of the viscosity effects of the fluid [118].

Satellite particles were observed during the fast gelation process following the main particles. They modified the channel configuration by using two adjacent channels joining at the outlet for producing Janus particles (Figure 10E,F). Because the droplets traveled the air gap with high speed and immediately became crosslinked after entering into CaCl_2_, the diffusion and convection between the hemispheres were reduced, allowing the reservation of two different hemispheres with a clear interface between them. They used red and green fluorescent beads mixed with Na-alginate solution for the production of Janus particles. Moreover, they produced biphasic Ca-alginate particles loaded with Fe_3_O_4_, which was an attractive vehicle for controlled delivery systems in the biological field; the hemisphere containing magnetic materials could be manipulated by an external magnetic field while the other hemisphere could be used as a carrier of bioactive substances (Figure 10G–I).

**Figure 11:**
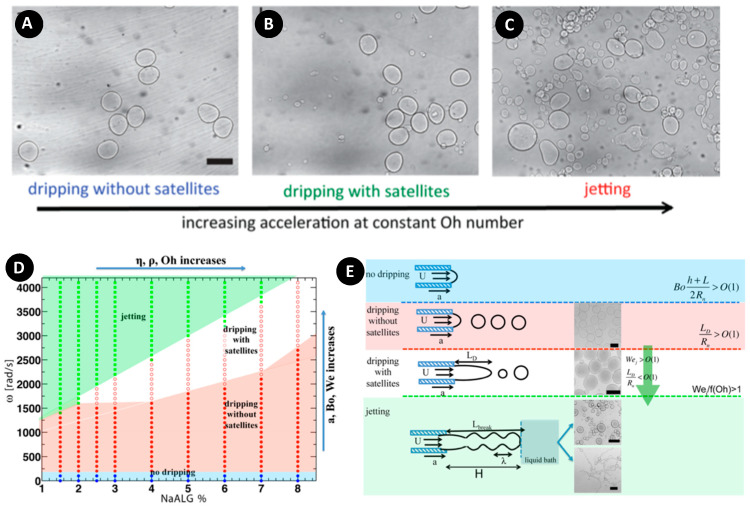
(**A**–**C**) Microscope images of produced particles at different acceleration corresponding to different regimes. (**A**) Dripping without satellites; (**B**) Dripping with satellites; and (**C**) Jetting; (**D**) Experimental phase diagram showing different break-offs of droplets as a function of rotational speed and Na-alginate concentration; (**E**) Illustration of different pinch-off regimes. Microscope images of particles at different accelerations. Reprinted with permission from [69].

Previously, scientists have struggled to produce monodisperse particles and had many trials and errors. Eral et al. improved the overall physical understanding of the centrifugal hydrogel formation by taking into account the pinch-off phenomenon for accurately predicting the average size of hydrogels in dripping without satellites regime [69]. They studied the emergence and transition of different pinch-off regimes (dripping without satellite (Figure 11A), dripping with satellite (Figure 11B), and jetting (Figure 11C)), which provided helpful information for experimentalists. Their results indicated that some experimental parameters affecting the pinch-off regime and particle size were not studied systematically in the previous studies [42,119]. Some of these parameters are contact angle, fluid viscosity, needle length, fluid level, and the distance between the nozzle tip and crosslinker solution. A comparison of the experimental results with the estimation of hydrogel size by equations, found that the equation numerically validated by Yildirim [120] provided a better fit than Tate’s law [69]. Accordingly, it was proposed that Tate’s law ignored the pinch-off process for estimating the average particle size. The results hypothesized that the volume of the pinched-off droplet was smaller than the pendant droplet because, in the pinch-off process, a portion of the droplet stayed behind. Therefore, it was concluded that the pinched-off process needed to be taken into account in equations. In addition, since droplets could shrink–swell their size upon crosslinking, they modified Yildrim’s equation by introducing a swelling/shrinking parameter C in Equation (9) to take into account the material properties affecting the size of droplet after crosslinking. The parameter C had to be determined prior to the experiment for the shrinking/swelling material in a simple dripping experiment for accurately predicting the particle size.

(9) $${\left(\frac{R_{p}}{R_{n}}\right)}^{3}=0.9372C^{3}Bo^{-1.07}$$

where *Bo* is the bond number, *R_p_* and *R_n_* are particle and nozzle radius, respectively.

Moreover, phase diagrams and scaling arguments based on the dimensionless parameters were provided to allow experimentalists to correctly choose operating conditions to achieve a monodisperse particle population size (Figure 11D,E). For most liquids right after the dripping to jetting transition, the liquid column fragments due to the inertia of the jet impact to the bath. It led to the pearl-necklace geometries in high Na-alginate concentrations. In low Na-alginate concentrations, the jet broke up easily, and since the fragmentation dynamics were faster than the gelation process, it resulted in polydisperse size distribution.

**Figure 12:**
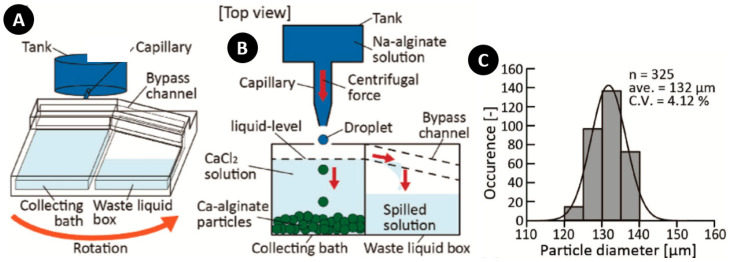
(**A**,**B**) Conceptual illustration of oil-free production of monodisperse Ca-alginate particles with a by-pass channel and waste chamber for transferring spilled solution in order to keep the CaCl_2_ solution level constant; (**C**) Diameter distribution of Ca-alginate particles. Reprinted with permission from [121].

Morimoto et al. produced oil-free Ca-alginate particles with a device containing two reservoirs for mass production purposes [121]. Since the liquid level rose in the particle gathering chamber as more droplets entered into it, polydispersity increased because of the non-constant distance between the nozzle tip and fluid level; additionally, clogging of the capillary occurred as the level of the fluid approached the nozzle tip. To avoid this, they introduced a waste chamber connected to a gathering reservoir with a bypass channel to keep the crosslinker level constant during particle generation (Figure 12A,B). Moreover, they showed the capability of cell-laden alginate particles by producing Ca-alginate particles encapsulating NIH3T3 cells and covered with NIH3T3 cells. Particle containing cells had an average diameter of 165 µm and a CV of 2.68%, and showed a good monodispersity (Figure 12C). Although some cells died because of shear stress of Na-alginate in rotating, the cell viability study showed that 94% and 85% of the cells were alive after 1 h and 1 day of encapsulation, respectively. To investigate the possibility of covering Ca-alginate particles with NIH3T3 cells as a cellular module for constructing macroscopic three-dimensional (3D) cellular structures, they assembled Ca-alginate particles in a human doll-shaped PDMS mold. The results showed that after one day of culture in the PDMS mold, the cells on the particles adhered to cells on the other particles. The importance of this method lies behind the rapid fabrication process (240 s) for the construction of the particles as compared with previous works, which needed 1 h of fabrication process for the same human-doll shape [122].

We have summarized the important characteristics of dispenser nozzle studies in Table 2. These characteristics include diameter range, CV, nozzle size, and rotational speed/acceleration.

### 6.3. Crossflow Method

**Figure 13:**
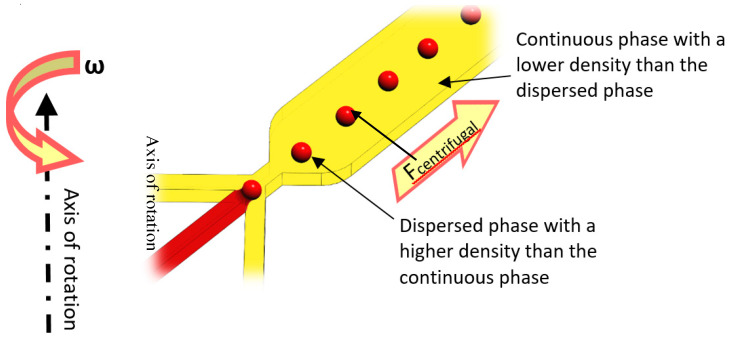
A schematic view of the crossflow mechanism of droplet generation on lab-on-a-disk (LOD) devices.

Using shear force to produce droplets is a common approach in the literature, specifically for LOC devices. According to Figure 13, in the case that the droplets’ density is higher than the continuous phase’s density, the phenomenon would be similar to the conventional T or +junctions. In centrifugal setups, the drag force of sheath flows and centrifugal force pull the dispersed phase to break off and form droplets. Droplet size can be adjusted by the channel geometry and rotational speed. Different geometries and materials can be used to produce droplets by this method.

**Figure 14:**
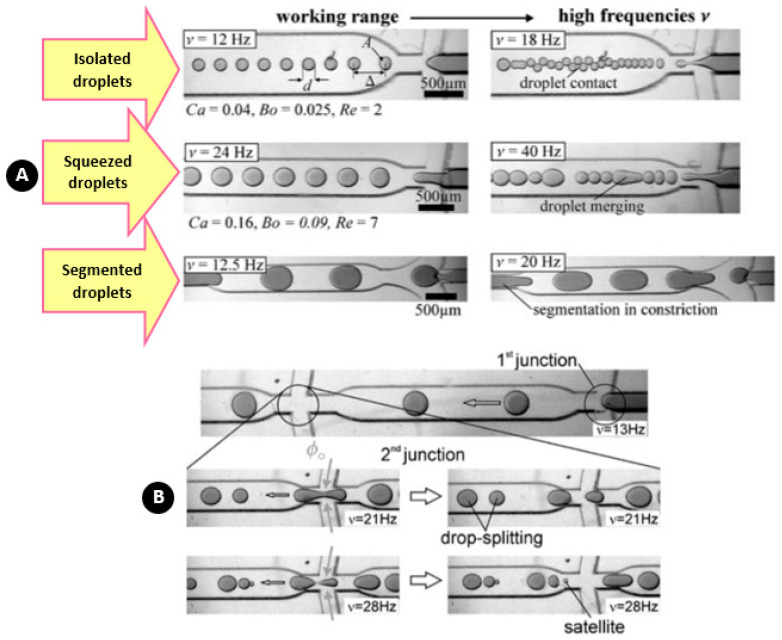
(**A**) Identification of three droplet structures depending on the channel geometry and rotational frequency; (**B**) Droplet splitting at the downstream in different frequencies. Reprinted with permission from [46].

For the first time, Haeberle et al. used the centrifugal force to generate droplets with cyclo-olefin copolymer (COC) substrate [46]. To avoid the jetting regime, they throttled the disperse phase flow by increasing hydrodynamic resistance of the main channel in order to reduce the capillary number for supporting the desired dripping regime. The flow rates were dynamically affected by angular velocity and statically by channel resistances. According to Figure 14A, they categorized the multiphase flow into three different structures of “isolated droplet”, “squeezed”, and “segmented”. They identified that within a certain window of rotational speeds, highly monodisperse droplet trains were generated for all the three droplet structures. The results related to the droplet generation rate indicated that isolated droplets have a greater tendency for high-throughput capability as compared with the squeezed flow regime. Moreover, as shown in Figure 14B, they used a secondary junction to split the generated droplets into sub-droplets of a defined size. They reported that in low rotational frequencies, the droplets were transiently squeezed, and then relaxed back to their equilibrium shape right after the splitting junction. Around the frequency of 21 Hz for their specific work, two spatially separated droplets were formed at the downstream. At high rotational frequencies, the isolation of satellite droplets was reported because of reinforced flow-focusing at the junction. Droplet sedimentation, based on the density difference, was also carried out in their work. The flow-splitting structure was comprised of a slanted arm toward the centrifugal force which pushed the droplets toward the outer wall within the guide channel until leaving the first outlet. They also produced two-phase droplets by guiding pure water and ink to the junction of the flow focusing structure. These droplets could be useful for facilitating mixing in very small reaction chambers [123,124] or they could be turned into Janus particles by polymerization methods, which would be applicable in display technologies [125,126].

**Figure 15:**
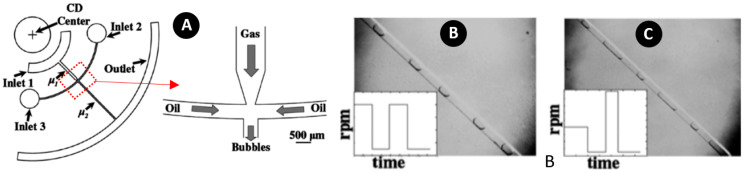
(**A**) Gas-bubble formation configuration; (**B**,**C**) Bubble generation by changing rotational speed to form various sizes with different intervals. Reprinted with permission from [127].

This method was also utilized for gas-bubble generation. For the first time, Chakraborty et al. produced gas bubbles inside silicon oil using a cross-junction on a centrifugal platform (Figure 15A) [127]. The high viscosity difference between the gas and oil, made a sharp transition from dripping to jetting regimes. To overcome this constraint, they used variable rotational speeds to open up a broader range of frequencies and bubble dimensions. Following this strategy, as shown in Figure 15B,C, the disc was first rotated at high speed and kept rotating for a specific time to put the flow into the centrifugal dominated regime. This way, they could create a long stream of jet gas. Then, the rotational speed was suddenly decreased to let the surface tension dominant, and trigger the bubble snap-off process. In other words, increasing and decreasing the rotational speed made the bubble formation possible.

**Figure 16:**
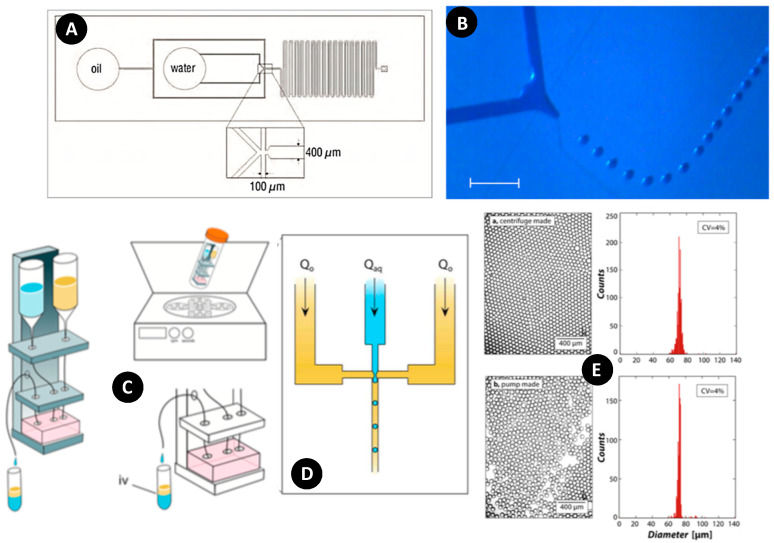
(**A**) Configuration of droplet generation on a centrifugal microfluidic platform; (**B**) Water-in-oil droplet generation at 550 rpm the system. Scale bar 400 µm. Reprinted with permission from [55]; (**C**) Schematic illustration of droplet generation with syringe reservoirs; (**D**) Flow focusing configuration platform for droplet generation; (**E**) A comparison of monodispersity between the results of a syringe pump (lower figure) and centrifugal system (upper figure). Reprinted with permission from [62].

Later, experimental tests of Hugo et al. showed the capability of producing monodisperse droplets on disc with high stability [55] (Figure 16). In their experiment, a crossflow design with 100 µm inlets and a 400 µm outlet was built using PDMS (Figure 16A), and then mounted to a PMMA rotating disc to generate droplets (Figure 16B). Limited reservoir capacity and difficulty in applying and adjusting initial pressure at the inlets were some limitations of centrifugal microfluidics platforms. One year later, Ahmed et al. designed an innovative method for addressing these issues [62]. They used falcon tubes as reservoirs and connected them to the inlets and outlets of the microfluidic device. A scaffold was built, and the entire system, including falcon reservoirs, microfluidic devices, and connecting tubes, were launched on it to rotate (Figure 16C). As depicted in Figure 16D, a crossflow configuration was used for droplet generation. In addition to adjusting the rotational speed as a conventional control method of centrifugal microfluidic systems, changing the height of reservoirs and inlet resistance provided agility, ease, and flexibility for producing highly monodisperse droplets. Moreover, they demonstrated the applicability of this method for cell encapsulation and culture assays. As represented in Figure 16E, a monodispersity comparison was made between the droplets generated by a syringe pump and centrifugal system; the results indicated good concurrence.

We summarize previous crossflow studies in Table 3. These characteristics include diameter range, CV, and rotational speed/acceleration.

### 6.4. Other Methods

**Figure 17:**
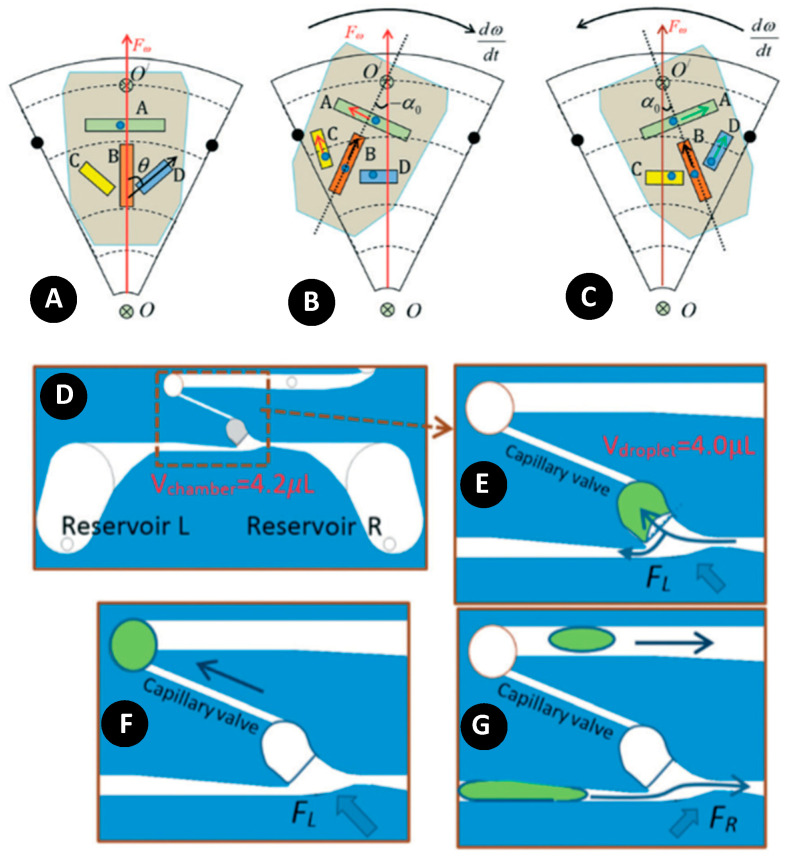
(**A**–**C**) The dynamics of flow in four different channels inside binary chip; channel A is azimuthal, channel B is diagonal, channels C and D are slanted; (**A**) Initial state; (**B**) LEFT state; (**C**) RIGTH state. (**D**,**E**) Producing droplet with binary chip. (**D**) Design of a binary chip with two reservoirs at the left and right, a metering chamber, a capillary valve, and a main channel; (**E**) In the LEFT state, the metering chamber is filled with liquid and the extra amount is directed to the left reservoir; (**F**) Increasing the rotating speed, the droplet breaks the capillary valve and enters to the main channel; (**G**) the extra amount of the liquid in the left reservoir returns back to the right reservoir. Reprinted with permission from [28].

Some other creative methods have been used in the literature to produce droplets in centrifugal microfluidics. Wang et al. made a new binary design in which the direction and amount of centrifugal force were changed inside the channels during operation [28]. As shown in Figure 17A–C, manipulating the position of the binary chip (initial, LEFT, or RIGHT state) by modifying the angular acceleration changed the centrifugal force direction inside the channels. The basic principle was inspired by an auto fish feeder, in which the setup consisted of two conjugated reservoirs, a metering chamber, and a capillary valve (Figure 17D). As shown in Figure 17E, the channel for filling the metering chamber had a smooth arch shape at the junction, while the other side of the channel had a very sharp angle. In the LEFT state, the metering chamber was filled and the rest of the liquid went to the left reservoir (Figure 17E). As depicted in Figure 17F, by increasing the rotating speed to 1200 rpm, the capillary valve broke and the droplet was released to the main channel. By switching the chip to the counterpart state, the centrifugal force was in the right-up direction, and the redundant sample in the left reservoir moved back to the right reservoir (Figure 17G).

**Figure 18:**
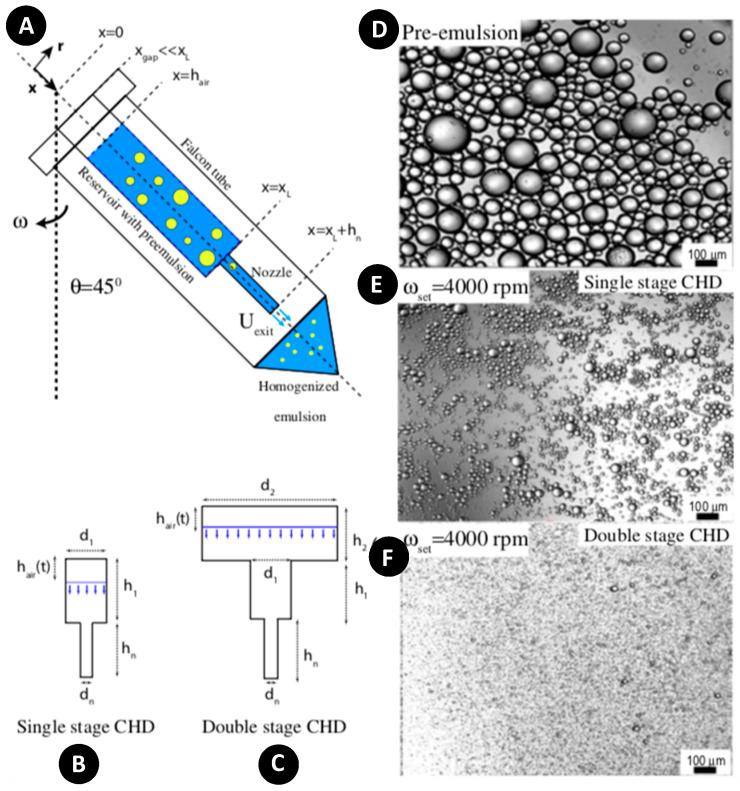
(**A**) Schematic of centrifugal homogenization device (CHD) in which a 20 mL syringe used as a reservoir and a nozzle dispenses the emulsions into the falcon tube cap. The whole system rotates at an angle of 45° to the rotation axis; (**B**) Schematic of the single-stage CHD with 2 mL reservoir; (**C**) Schematic of the double-stage reservoir with two reservoirs, i.e., a 2 mL and another 20 mL; (**D**–**F**) Microscope images of coarse pre-emulsion and resulting emulsions after homogenization at 4000 RPM and repeating the process 5 times. Reprinted with permission from [129].

The centrifugal homogenization process is another way of generating monodisperse droplets. Singh et al. studied theoretical modeling and experimental setup of a centrifugal homogenization for laminar and turbulent setups [129]. They demonstrated the successful application of a centrifugal homogenization device (CHD) in both emulsification and mechanical cell lysis. In the single-stage CHD configuration (Figure 18A,B), a 2 mL plastic syringe reservoir was connected to a needle and fitted inside a 50 mL falcon tube as an emulsion collector. In the double-stage device, another 20 mL sample bottle was used as the second wider reservoir (Figure 18C). The entire system was slanted at an angle of 45° to the vertical axis of the centrifugal rotor. For emulsification with this device, pre-emulsion was prepared and inserted into the reservoir, and then the whole system rotated. The dependence of emulsion droplet size on centrifugal speed, nozzle size, and dispersed phase viscosity were studied. The results showed that the double-stage CHD improved the homogenization performance and produced smaller droplets with narrower size distributions as compared with the single-stage CHD (Figure 18E,F). Furthermore, because the designed device could provide a high power density, which was comparable to previous works in the literature [130], it could be used to mix high viscosity fluids and breakup of aggregates. However, the time-dependent behavior of the liquid volume height and rotational speed led to a non-uniform shear rate and, consequently, increased the polydispersity of the resulting emulsion.

**Figure 19:**
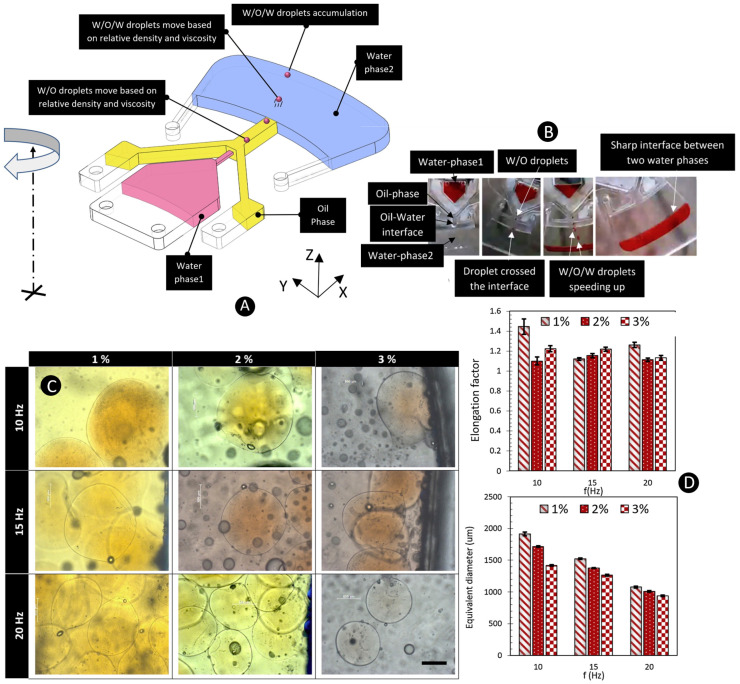
(**A**) The schematic of fluidic barrier concept; (**B**) The sequential images of the production of double emulsion on centrifugal microfluidics; (**C**) Microscope images of the particles using different frequencies and alginate concentrations. The scale bar is 500 μm; (**D**) Aspect ratio and diameter of the generated particles as a function of rotational frequency and alginate concentration. Reprinted with permission from [131].

Recently, Madadelahi et al. (2020) combined two different methods, i.e., step emulsification and dispenser nozzle, to make alginate microspheres [131]. Moreover, for the first time, they were able to make double emulsions on centrifugal platforms. Figure 19A shows this method called “fluidic barrier.” As depicted in the schematic figure, the first liquid (water phase1 shown in red) entered the second liquid (oil phase shown in yellow), and then the red droplet covered with a layer of yellow liquid entered the next liquid media (water phase2 shown in blue). As shown in Figure 19B, double emulsion generation of water-in-oil-in-water was successfully demonstrated. Then, they changed the materials to produce alginate particles. Figure 19C shows the microscope images of generated particles. Three different frequencies of 10, 15, and 20 Hz and three different alginate concentrations of 1%, 2%, and 3% were considered. Figure 19D shows the diameter and aspect ratio of the particles and indicates that the higher the frequency, the smaller the particles are. As shown, the higher the frequency, the smaller the particles. The same is true for the relation between the alginate concentration and the size of particles. However, as shown, the aspect ratio was not a linear function of rotational speed.

## 7. Comparison of Different Centrifugal Methods

In contrast to non-centrifugal microfluidics platforms, parallelization is easily and inexpensively applicable to all methods in centrifugal systems and, in a reliable manner, can accelerate the droplet/particle production rate considerably. In Table 4, we summarize the process parameters and resulting droplet/particle characteristics of reported works in a way that readers can find the contrasts easily.

### 7.1. Step Emulsification

Buoyancy has a significant influence in droplet formation in the step emulsification method, and it helps to increase the droplet generation rate while preserving monodispersity. Additionally, buoyancy helps to pack the produced droplets after formation and release the excess continuous phase to achieve high-volume fractions. Moreover, because the design for this method is simple, fabrication techniques are easy and inexpensive. Therefore, for fast and easy production of high-volume fraction emulsions, the step emulsification method is the right choice.

### 7.2. Dispenser Nozzle

Similar to the step emulsification method, in the dispenser nozzle method, the continuous phase is only kept at the storage chamber; therefore, the need for a continuous phase is minimal. Because of the changing distance between the nozzle tip and the continuous phase solution during operation, additional configurations ensuring a constant level of reservoirs should be made for reliable and monodispersed production of particles. Since, in most cases, droplets travel through an air gap, and then enter to the continuous phase, this method is relatively sensitive to the material choice to keep the shape of droplets stable. In the literature, this method has mostly been used for the production of polymer particles. According to Table 4, the produced particles are comparatively bigger with higher CV and lower production rates.

### 7.3. Crossflow

In contrast to step emulsification and dispenser nozzle methods in which the continuous phase is stationary, and the consumption rate is low, in the co-flow method the continuous phase is flowing along with the dispersing phase; therefore, this method uses relatively higher amounts of continuous phase to make the pinch-off process possible. Additionally, adjusting proper flow rates for dispersing and continuous phases at the same time on a rotating disc with just one variable (rotating velocity) is challenging for the production of monodisperse droplets.

### 7.4. Other Methods

Depending on the underlying mechanism for the production of droplets in centrifugal systems, production rate, and monodispersity can vary. For example, in a binary droplet generation unit [28], the droplets are metered before releasing into the main channel; thus, it can produce highly monodispersed droplets. However, since filling the metering chamber and breaking the capillary valve necessitate several switching states, on the one hand, the production rate is much lower. On the other hand, the centrifugal homogenization device [129] can produce emulsions in a large quantity in a short period of time but by compromising monodispersity.

## 8. Commercialization

As mentioned previously, all LOD devices can benefit from the advantages of multiphase operation units. Until 2015, at least 15 LOD devices passed the final steps to become commercially available [132]. Now, we can still see that the commercialization of LOD technology has been surprisingly difficult and slow. The most mature and proven player remains Abaxis with its Piccolo^®^ blood chemistry analyzer [133]. A second runner up, in terms of maturity and established sales, with technology originally developed at Samsung, is NexusDx’s IB10 immuno-analyzer that quantitatively measures in-vitro antigens or antibodies in whole blood or plasma samples on dedicated test discs. Another contender is GenePOC with a molecular diagnostic test on LOD that generated total revenues of less than one million USD in 2018. In 2019, Meridian Bioscience signed an agreement to acquire Canadian GenePOC in a deal valued at 120 million USD [134].

Interestingly, the current COVID-19 pandemic is accelerating the commercialization of products based on LOD technology. Tsinghua University announced the delivery of Corona virus tests to Wuhan Hospitals with a system based on LOD technology [135]. Similarly, the test system of Spindiag GmbH for proof of the coronavirus Sars-CoV-2 in 30 to 40 min is predicted to be available as early as the third quarter of 2020 [136].

## 9. Conclusions and Outlook

This paper has summarized and compared different techniques of particle, bubble, droplet, and fiber generation on centrifugal microfluidic devices. We classified these methods of dispenser nozzle, step emulsification, and crossflow techniques. We discussed all specifications according to the previously available studies in the literature. Alongside the theoretical aspects, we also covered the comparison of these methods in detail, such as diameter range, dispersity of products, production rate, rotational speed rate, and also pros and cons of each method. Among the studied methods, step emulsification was found to be a suitable method in terms of fast production rate, simple fabrication, and low continuous phase consumption. In addition, it can produce droplets/particles with high monodispersity and high volume-fraction by exerting low shear force during the fabrication process. The dispenser nozzle method can provide an air gap to prevent the direct contact of dispersed and continuous phases, offering a suitable technique to avoid clogging problems. The fluidic barrier, as a combination of dispenser nozzle and step emulsification, seems to be a rewarding method. The need for independently controlling more than one flow rate makes the crossflow configuration relatively hard to implement on centrifugal platforms. Additionally, the crossflow method uses large amounts of continuous phase to pinch off the dispersing phase. Other innovative methods in the literature indicate the high capability of centrifugal systems for droplet/particle generation.

## Figures and Tables

**Table 1:** Summary of reported step emulsification studies.

Material			Diameter Range	Volume Fraction	CV	Production Rate	Rotating Acceleration/Frequency	Ref.
Dispersed Phase	Continuous Phase	Surfactant						
-	Novec 7500	Picosurf-1 2% *w*/*v*	120~170 μm	97.2% at 20 Hz	2–4%	>500 Droplets per second per nozzle	5~40 Hz	[31]
Water, inkjet printer ink in water (10% *v*/*v*) and low melting agarose (1.25% *w*/*v*).	Novec 7500	Picosurf-1 2% *w*/*v*	156 μm	97.2~99%	4%	3700/s with 72 nozzles	10~20 Hz	[109]
Water	Novec 7500	Picosurf-1 5%	100 μm	-	<5%	2800/s per nozzle	1~100 Hz	[47]
-	Silicone oil, Fluorinert, Bio-Rad	-	103~200 μm	6~14%		-	8.3~41.7 Hz	[113]
-	Fluorinated oil	-	147 μm		1.4%	11,000	0.5~10 Hz	[111]
Water	Mineral oil containing 2 wt% Span 80	-	18 to 90 μm, ave 23.1 μm	-	1.6%	-	150~1000 g	[73]

**Table 2:** Summary of reported dispenser nozzle studies.

Material		Diameter Range (µm)	Nozzle Size (µm)	CV (%)	Production Rate	Rotating Acceleration/Frequency	Ref.
Dispersed Phase	Continuous Phase						
2% (*w*/*w*) chitosan	10% (*w*/*w*) TPP solution at pH 4.0.	257~148	127	22~15	0.7~45.2 per second	93~452 g 20~44 Hz	[115]
2~6% w Na-alginate	CaCl_2_	800~180	127	16~7	Up to 600 per second and channel	5~28 Hz	[42]
Aqueous solution (PCR mixture)	A binary mixture of 93% (*v*/*v*) isopropyl palmitate and 7% (*v*/*v*) ABIL EM180	165~45	6.2	11~3.4	-	1000~15,000 g	[64]
1%, 1.5%, and 2% (*w*/*v*) Na-alginate	2.5~20 wt% CaCl_2_ (0.5% Tween 20) for surfactant	269~109	Depth 40~80 Width 100~1000	5.6~5.2	-	130~515 g	[79]
1.5~8% w Na-alginate	CaCl_2_	-	80		-	1~500 g	[69]
Calcium carbonate (CaCO_3_)	CaCl_2_	150	-	4~2.7	170 droplet/s	350 g	[121]

**Table 3:** Summary of reported crossflow studies.

Material		Diameter Range (µm)	CV (%)	Rotating Acceleration/Frequency	Ref.
Dispersed Phase	Continuous Phase				
Air	Silicone oil	-	-	variable	[127]
Water	Sunflower oil	270~188	-	14.3~27 Hz	[63]
Water	Mineral oil	80	-	9.2 Hz	[55]
Water	Sunflower oil	211	2	7~33 Hz	[46]
Water	Silicon oil	-	-	3.2~23.9 Hz	[104]
Water	HFE-7500 oil	-	-	3.3~15.8 Hz	[62]
Water	Silicon oil	82~90	-	3.3~50 Hz	[128]

**Table 4:** Comparison of different methods of droplet/particle generation according to the previous studies in the literature.

Characteristics		Step Emulsification	Crossflow	Dispenser Nozzle	Other Methods		
					Binary Unit	Centrifugal Homogenization	Fluidic Barrier
Diameter Range (μm)		18~200	80~270	45~800	7700	18~35	280~1900
CV%		1.4~14	2	2.7~22	-	20~50	-
Production Rate (per second per nozzle)		50~2800	-	0.7~600	-	-	0.003~1
Rotating Criteria	Speed (Hz)	0.5~100	3.2~50	5~44	5~20	50~100	10~20
	Acceleration (m/s^2^)	150~1000 g	-	1~15,000 g	-	-	-
Density sensitive		Yes	No	Yes	No	No	Yes
Limitations		☑Highly density dependent (i.e., limited materials)	☑High value of shear forces ☑High consumption of the continuous phase ☑The need of adjustment of different flow rates	☑Limited material selection ☑Formation of tail and non-spherical particles	☑Low production rate	☑Low monodispersity	☑Highly density dependent (i.e., limited materials)
Strength		☑High monodispersity ☑Fast production rate ☑low shear force ☑Simple fabrication ☑High volume-fraction emulsions ☑Low need of continuous phase fluid	-	☑Low need of continuous phase fluid	☑High monodispersity	☑High production rate ☑Simple fabrication method	☑Low shear force ☑High volume-fraction emulsions ☑Low need of continuous phase fluid
Other remarks		-	-	☑This method is mostly used for particle generation, not droplets	-	-	☑This is a general method applicable to all different methods for generation of multiple emulsions and microparticles
Applications		Nucleic acid amplification (ddRPA) [31] Gel emulsion for pharmaceutical, food, and homecare products [109] ddPCR; cell-based assays [47,111] POC [113] Picolitre compartments for biochemical assays, preparation of cell-sized functional microbeads [73]	Food, cosmetics, controlled release of chemicals and drugs, imaging, heavy metal removal during mineral processing, development of bubble-based logic circuits, and [46,127,128] PCR, hydrogel engineering, optical sensors and scaffolds for living tissue [63] POC [55] Multiplexing diagnostic assays; cell encapsulation and separation, culture-based screens, dPCR, and digital ELISA [62,104]	Cell, proteins, enzymes and drug encapsulation and their controlled release [42,79,115] dPCR [64] Applications ranging from medical diagnostics to anticounterfeiting technologies [69] Biomedical and tissue engineering; cell therapy and cell cultivation [121]	Complex bioassays such as the Bradford assay and DNA purification assay, (PCR) real-time DNA amplification [28]	Mechanical cell lysis (mechanical lysis of mpkCCD mouse kidney cells) [129]	Cell encapsulation, drug delivery, and digital PCR [131]
